# Long-distance control of the scion by the rootstock under drought stress as revealed by transcriptome sequencing and mobile mRNA identification

**DOI:** 10.1093/hr/uhab033

**Published:** 2022-01-19

**Authors:** Marzieh Davoudi, Mengfei Song, Mengru Zhang, Jinfeng Chen, Qunfeng Lou

**Affiliations:** State Key Laboratory of Crop Genetics and Germplasm Enhancement, College of Horticulture, Nanjing Agricultural University, Weigang Street 13 No.1, Nanjing 210095, China

**Keywords:** Grafting, Transcriptome, Grafting Biology

## Abstract

Grafting with pumpkin rootstock is commonly used not only to improve the quality of cucumber fruits but also to confer biotic or abiotic stress tolerance. However, the molecular mechanism of the response of grafted cucumbers to drought stress and the possible roles of mobile mRNAs in improving stress tolerance have remained obscure. Hence, we conducted transcriptome sequencing and combined it with morphophysiological experiments to compare the response of homografts (cucumber as scion and rootstock) and heterografts (cucumber as scion and pumpkin as rootstock) to drought stress. After applying drought stress, homografts and heterografts expressed 2960 and 3088 genes, respectively, in response to the stress. The differentially expressed genes identified in heterografts under drought stress were categorized into different stress-responsive groups, such as carbohydrate metabolism (involved in osmotic adjustment by sugar accumulation), lipid and cell wall metabolism (involved in cell membrane integrity by a reduction in lipid peroxidation), redox homeostasis (increased antioxidant enzyme activities), phytohormone (increased abscisic acid content), protein kinases, and transcription factors, using MapMan software. Earlier and greater H_2_O_2_ accumulation in xylem below the graft union was accompanied by leaf abscisic acid accumulation in heterografts in response to drought stress. Greater leaf abscisic acid helped heterografted cucumbers to sense and respond to drought stress earlier than homografts. The timely response of heterografts to drought stress led to the maintenance of higher water content in the leaves even in the late stage of drought stress. The mobile mRNAs identified in heterografts were mostly related to photosynthesis, which would be the possible reason for improved chlorophyll content and maximum photochemical efficiency of photosystem II (Fv/Fm). The existence of some stress-responsive pumpkin (rootstock) mRNAs in cucumber (scion), such as heat shock protein (*HSP70*, a well-known stress-responsive gene), led to higher proline accumulation than in homografts. Expression of mobile and immobile stress-responsive mRNAs and timely response of heterografts to drought stress could improve drought tolerance in pumpkin-rooted plants.

## Introduction

Naturally, plants face various biotic and abiotic stresses during their life, like drought, high or low temperature, salinity, and so on [1]. Among them, drought is one of the most critical environmental limitations for plants, especially in arid and semiarid regions, and is expected to increase as a result of global climate change [2]. Generally, drought stress (WS) triggers a series of responses such as photosynthesis reduction, oxidative stress, producing an excessive amount of reactive oxygen species (ROS) [3], lipid peroxidation, and membrane damage, leading eventually to protein denaturation [4] and cell death [5]. Notably, variations in the expression of transcription factors, protein kinases, and stress-responsive genes are among the molecular responses to WS [6]. Cucumber (*Cucumis sativus* L.) is one of the most widely cultivated vegetables in the world, and has a shallow root system and wide leaves [7]. These characteristics make cucumber sensitive to water deficiency, and consequently its yield and quality can be negatively affected by WS as the drought period increases [8].

Some studies have shown that grafting cucumber with a tolerant rootstock can confer stress tolerance on sensitive scions [9]. Grafting is a simple, common, and environmentally friendly method that has been used for a long time. By this method, two individual plants with different traits can be joined together, one of them acting as a root system, called the rootstock, and the other forming the aerial part, called the scion [10]. This technique can be used for different goals, such as improving plant tolerance to biotic or abiotic stresses, increasing agricultural crop yields, and asexual propagation [11]. Pumpkin (*Cucurbita moschata*) is one of the most popular rootstocks among farmers in China and is used in the commercial realm not only to improve plant vigor and quality of cucumber fruits (glossy fruit) [12] but also to enhance tolerance to different environmental stresses, such as salt [13], drought [14], cold [15], and heavy metals [16]. This vigorous rootstock has been reported to induce stomatal closure in cucumber scions at the very early stage of salinity stress, which leads to reductions in transpiration rate and water loss under osmotic conditions [9]. Another study proposed that the pumpkin rootstock senses salt stress earlier than self-grafted cucumber and induces rapid closing of the stomata as a result of quick root-sourced hydrogen peroxide (H_2_O_2_) signaling and greater abscisic acid (ABA) accumulation, which leads to maintenance of the proper amount of water inside the plants under stressful conditions [17]. Similarly, luffa rootstock also increased the ABA sensitivity of rootstock-grafted cucumbers which, makes them strongly sense the water deficiency in the soil then induce stomatal closure, which leads to higher water use efficiency and improved drought tolerance [14]. A study proposed that there is a link between ABA, H_2_O_2_, and heat shock proteins (HSPs) to induce heat tolerance in cucumber grafted onto pumpkin [18]. HSP70 is one of the well-known molecular chaperons that protect plants against abiotic stresses [19] and some studies have shown the vital role of this chaperon in conferring stress tolerance [20]. Grafting studies have proposed that root-source signals such as ABA and H_2_O_2_ [17] could increase the HSP70 level in response to abiotic stresses [21], subsequently inducing proline accumulation and stress tolerance in plants [18]. Generally, rootstocks can induce constitutive (expressed under both normal and stress conditions) and drought-responsive (expressed only under stressful conditions) modifications in scions to improve drought tolerance [22]. For instance, grafting watermelon onto pumpkin rootstock improved water use efficiency and fruit yield under normal and WS conditions (constitutive response) [23]. In contrast, sometimes rootstocks play their positive roles only under stress conditions, such as improving the growth characteristics of grafted pepper plants under WS (drought response) [24].

Transcriptome studies revealed that grafting could influence gene expression in the scion, which may subsequently confer new traits or stress tolerance on the whole plant system [25]. A previous study showed that stress-responsive genes, transcription factors, phytohormones, and protein kinases were mainly down-regulated in tolerant citrus rootstocks in response to WS [26]. The higher number of differentially expressed genes (DEGs) in self-grafted watermelons than in squash-grafted plants can be attributed to homografts’ sensitivity to chilling stress [27]. In contrast, another study showed that ‘Rangpur’ lime as a tolerant rootstock mainly induced the expression of stress-responsive genes in sweet orange scions under WS. These findings suggested that the responses of plant species to the stress condition would vary based on genotype, the duration of exposure to the stress condition, and the intensity of the stress [26, 28].

One of the interesting facts about grafting is that the rootstock is capable of communicating with the scion by translocating mobile mRNAs (mb-mRNAs) under normal or stressful conditions [29]. The results of a recent study led to the identification of a large number of mb-mRNAs under chilling stress using watermelon and bottle gourd heterografts. The authors proposed that these mb-mRNAs, which were related to stress sensing, membrane stability, energy metabolism, and ABA signaling, made a shoot–root connection and led to cold stress tolerance in the scion [30]. The movement of mRNAs also has been reported under phosphate (P_i_) stress using cucumber and watermelon heterografts. The identified mb-mRNAs were grouped into four classes, comprising those enriched in translation and metabolism (groups 1 and 2), DNA regulation, and signaling (groups 3 and 4), which help plants to respond to stress efficiently. It was proposed that these mb-RNAs contribute to stress tolerance in scions [31].

As described above, this widespread and popular horticultural technique is widely used in the Cucurbitaceae family and many other plant species to improve stress tolerance. However, the molecular responses of grafted plants to WS have not been well studied. Accordingly, understanding the molecular responses of grafted plants to this environmental threat can help breeders and researchers have a better insight into mechanisms induced by the rootstock to alleviate the drought stress in sensitive scions and whole plants. Therefore, we designed an experiment to study the molecular responses of grafted cucumber to desiccation and investigate how the rootstock can affect gene expression in the scion under drought stress conditions.

## Results

### Morphological and physiological responses of homografts and heterografts to drought stress

**Figure 1:**
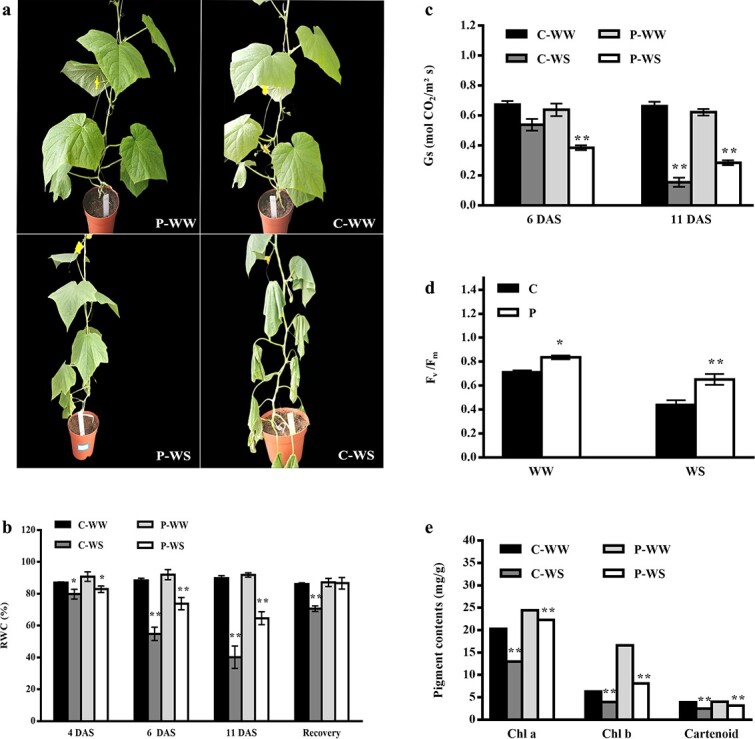
Pumpkin rootstock induces drought tolerance in cucumber scion. (**a**) Growth phenotypes of homografts (C) and heterografts (P) under well-watered (WW) and drought stress (WS) conditions. The photographs were taken at 11 DAS and soil water content was kept at 30%–35% of field capacity. (**b**) RWC of the leaf at 4, 6, and 11 DAS and 1 day after recovery. (**c**) Stomatal conductance (Gs) under WW or WS conditions at 6 and 11 DAS. (**d**) Maximum photochemical efficiency of PSII (Fv/Fm) at 11 DAS. (**e**) Chlorophyll a, b (Chl a, Chl b) and carotenoid contents at 11 DAS. The samples were taken from the topmost expanded leaves. Values are means of three replicates with standard deviations. Asterisks show significant differences according to Tukey’s test (^*^P < .05 and ^**^P < .01).

To investigate whether pumpkin as a rootstock is able to induce drought tolerance in cucumber scions, we made two types of grafted plants: homografts (cucumber as scion and rootstock) and heterografts (cucumber as scion and pumpkin as rootstock). Then, progressive drought stress was applied for 11 days. It is worth mentioning that the weighing method was used to maintain soil water content at a specific amount at each time point after drought stress (this is described in detail in the Materials and methods section). Comparison of homografts and heterografts showed that the leaves of cucumber-rooted plants were severely wilted at 11 days after drought stress (DAS) while pumpkin-rooted plants stayed more hydrated (Fig. 1a). Relative water content (RWC) of the leaves was examined at 4, 6, and 11 DAS and 1 day after recovery (rewatering). The results showed that the water content of the self-grafted plants sharply decreased, especially at 11 DAS. It is worth noting that heterografts maintained the highest leaf water content at all four time points compared with homografts. Heterografts were also able to strongly recover 1 day after recovery as there was no significant difference between recovered and control plants. Despite increasing the leaf water content of self-grafted cucumbers in response to recovery treatment, there was still a considerable difference between plants that had recovered from drought stress (C-WS plants) and the corresponding control (well watered; C-WW plants) (Fig. 1b). Then, we measured stomatal conductance, F_v_/F_m_, pigment (chlorophyll a, b, carotenoid) contents, and plant height of homografts and heterografts under both well-watered and stress conditions. Our results illustrated that the stomatal conductance of homografts did not change significantly while heterografts showed a remarkable reduction in stomatal conductance at 6 DAS (Fig. 1c). However, by increasing the exposure time to drought stress for 11 days, cucumber-rooted plants (C-WS) showed lower stomatal conductance compared with pumpkin-rooted plants (P-WS) (Fig. 1c). The use of pumpkin as rootstock notably increased photosynthesis pigments, F_v_/F_m_, and plant height (data not shown) under WW (control) conditions compared with self-rooted cucumbers. Despite decreasing these indices in both homografts and heterografts under drought stress, the heterografts (P-WS) maintained a greater amount of pigments and higher Fv/Fm than the homografts (C-WS) at 11 DAS (Fig. 1d and e).

### Determination of antioxidant enzyme activities, membrane integrity, starch and total soluble sugar (TSS) contents in homografts and heterografts in response to drought stress

**Figure 2:**
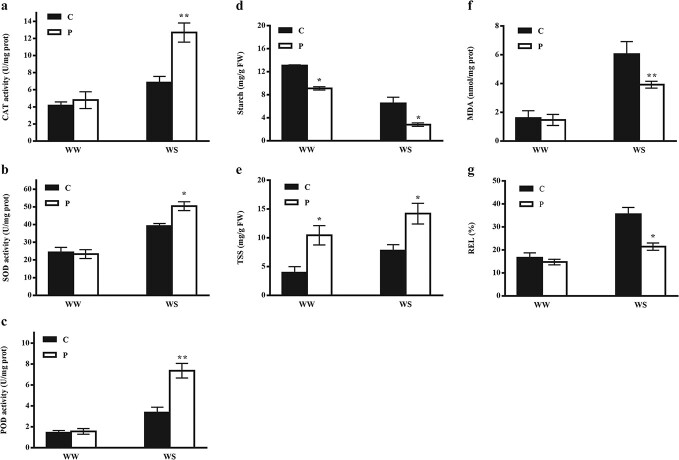
Influence of grafting and drought stress on antioxidant enzyme activity, starch and sugar content, and membrane integrity. All measurements were done at 11 DAS. Activities of (**a**) CAT, (**b**) SOD, and (**c**) POD in response to drought stress in homografts (C) and heterografts (P) under well-watered (WW) and drought stress (WS) conditions. (**d**–**g**) Starch (**d**) and total soluble sugar (**e**) contents, MDA (**f**), a lipid peroxidation product, and REL (**g**). Samples were taken from the topmost expanded leaves. Values are means of three replicates with standard deviations. Asterisks show significant differences according to Tukey’s test (^*^P < .05 and ^**^P < .01).

The activity of catalase (CAT), superoxide dismutase (SOD), and peroxidase (POD) increased significantly in pumpkin-rooted plants in response to drought stress (at 11 DAS), while there was no significant difference between homografts and heterografts under well-watered control conditions (Fig. 2a–c). We also measured the TSS and starch contents as two crucial energy sources, especially under abiotic stresses. The results indicated that P-WW contained a higher amount of TSS compared with C-WW. In response to WS, the sugar content increased in both homografts and heterografts compared with their corresponding control. However, P-WS showed the highest amount of TSS. Unlike TSS, homografts (C-WW) contained a greater amount of starch than heterografts (P-WW) which significantly decreased under WS conditions. In other words, heterografts contained a lower amount of starch than homografts not only under WW but also under WS conditions (Fig. 2d and e). We also examined relative electrolyte leakage (REL) and malondialdehyde (MDA, a lipid peroxidation product) contents as two main indicators of membrane integrity. The results revealed that there were no significant differences between P-WW and C-WW. However, WS caused a remarkable increase in REL and MDA contents in homografts, while heterografts showed a considerable reduction compared with homografts (Fig. 2f and g).

### Abscisic acid and hydrogen peroxide accumulate in leaf and xylem in response to drought stress

**Figure 3:**
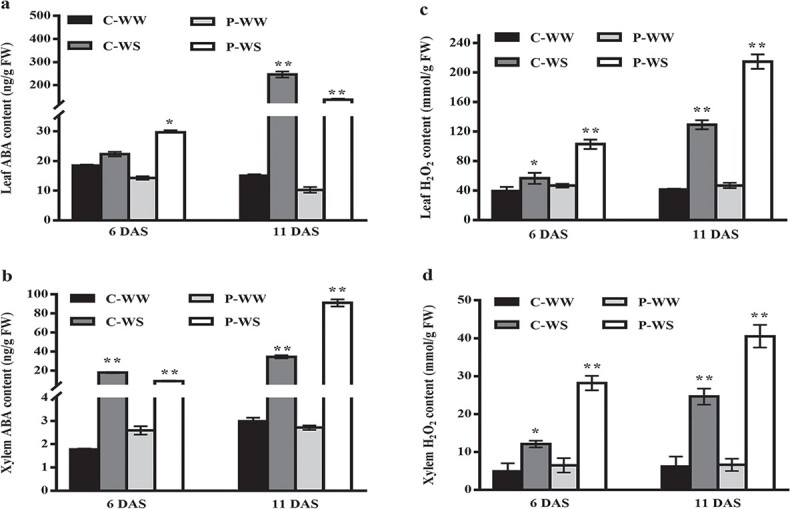
ABA and H_2_O_2_ accumulate in the leaves and xylem in response to drought stress. (**a**, **b**) ABA content in leaves (**a**) and xylem tissue (**b**) of homografts (C) and heterografts (P) under well-watered (WW) and drought stress (WS) conditions. (**c**, **d**) H_2_O_2_ content in leaves (**c**) and xylem (**d**). Leaf samples were taken from the topmost expanded leaves at 6 and 11 DAS. Xylem samples were taken from below the graft union. Values are means of three replicates with standard deviations. Asterisks show significant differences according to Tukey’s test (^*^P < .05 and ^**^P < .01).

Previous studies have shown the involvement of ABA and H_2_O_2_ in long-distance signaling and abiotic stress tolerance [18, 32]. Therefore, we examined whether ABA or H_2_O_2_ content is connected with pumpkin-induced drought tolerance. Our results revealed that leaf ABA content increased significantly in heterografts (but not in homografts) at 6 DAS. Interestingly, xylem ABA content significantly accumulated in both homografts and heterografts compared with their corresponding control at 6 DAS. The increasing trend of leaf ABA continued to 11 DAS, which led to late ABA accumulation in the leaves of homografts. Briefly, heterografts showed earlier leaf ABA accumulation (at 6 DAS), while this accumulation occurred in homografts at the late stage of WS (at 11 DAS). Xylem ABA content was also enhanced in homografts and heterografts in response to WS (at 6 and 11 DAS) (Fig. 3a and b). However, unlike leaf ABA, xylem ABA had higher accumulation in heterografts at 11 DAS than homografts. Leaf H_2_O_2_ increased in both homografts and heterografts at 6 DAS compared with their corresponding controls; however, leaf H_2_O_2_ content was more significant in heterografts. Leaf xylem showed an increasing pattern of H_2_O_2_ accumulation similar to that of leaf H_2_O_2_. Our findings also illustrated higher H_2_O_2_ accumulation in both leaf and xylem of heterografts in response to 11 days of WS (Fig. 3c and d).

### Transcriptome sequencing, assembly, and mapping

Transcriptome sequencing was used to compare the gene expression modifications in homografts and heterografts in response to WS. For this purpose, 12 libraries were made from leaf samples collected from P-WS, P-WW, C-WS, and C-WW plants. The prepared libraries of homografts and heterografts under WW and WS conditions were sequenced with an Illumina Novaseq sequencer, and 150-bp paired-end reads were generated. A total of 92.58 Gb of high-quality reads, ranging from 6.3 to 9.38 Gb for each sample, with an error rate of 0.03% and Q20 >96%, were produced. The clean reads were compared and aligned against the ‘Chinese Long v2’ cucumber reference genome (http://cucurbitgenomics.org) using HISAT2 software.

### Identification of differentially expressed genes in different comparison combinations

**Figure 4:**
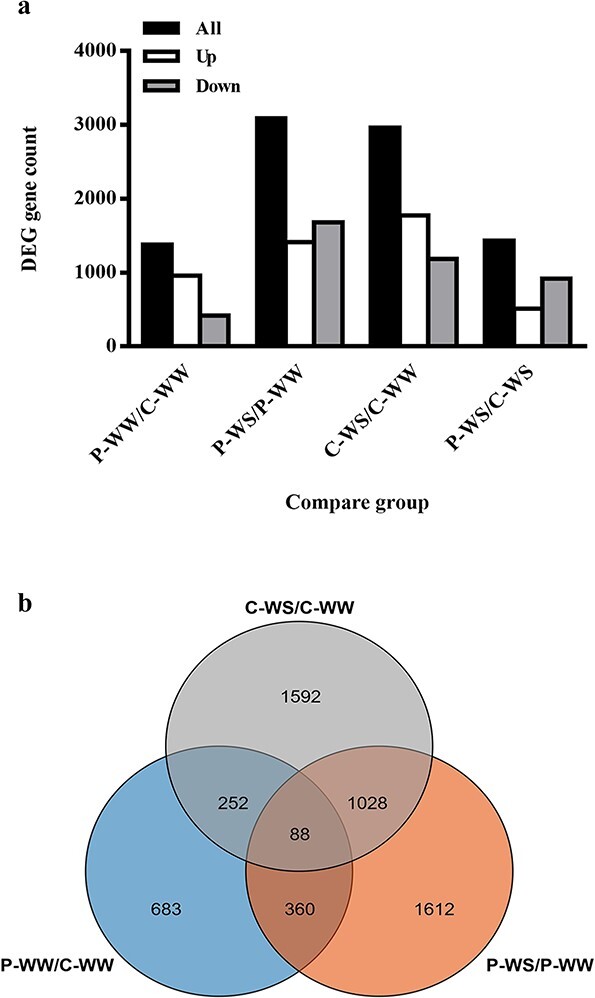
Number of DEGs among four treatment combinations. (**a**) Number of all DEGs in four treatment combinations, (**b**) Venn diagram showing the numbers of exclusive and common DEGs for all comparison combinations.

In the current study, we applied two treatments—plant grafting and drought stress—then performed transcriptome sequencing to compare the responses of grafted plants to WS (comparing P-WS/P-WW with C-WS/C-WW or direct comparison of P-WS with C-WS). Comparison of heterografts and homografts (P-WW/C-WW) showed that the expression level of 1383 genes changed in response to grafting with pumpkin rootstock, and the number of up-regulated genes (962 DEGs) was more than twice that of down-regulated genes (421 DEGs) (Fig. 4a). There was a large number of grafting-responsive genes (1383 DEGs) and most of them were up-regulated in response to the grafting treatment, indicating the significant influence of pumpkin rootstock on gene expression level in cucumber scion. As shown in Fig. 4, the highest number of DEGs was found in P-WS/P-WW (3088 DEGs), in which there were more down-regulated genes than up-regulated ones. Unlike P-WS/P-WW, in homografts (C-WS/C-WW) the number of up-regulated genes (1772 out of 2960) was higher than that of down-regulated ones. Furthermore, we could identify WS-responsive DEGs that were specific to heterografts and homografts using a Venn diagram. Our results showed that there were 1612 and 1592 DEGs specific to heterografts and homografts under WS, respectively (Fig. 4b). Interestingly, 360 of the grafting-responsive genes (P-WW/C-WW) were also present in P-WS/P-WW, showing the potential roles of grafting-responsive genes in WS.

### Functional annotation of the identified differentially expressed genes

**Figure 5:**
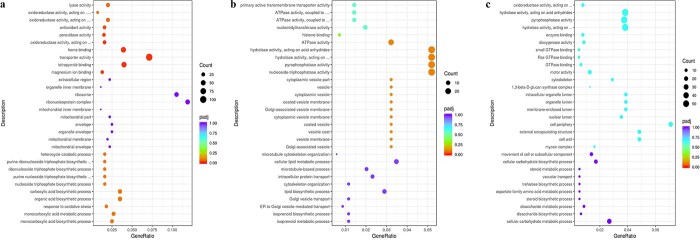
GO enrichment analysis of DEGs. Top 30 GO terms for the identified DEGs in (**a**) P-WW/C-WW, (**b**) C-WS/C-WW, and (**c**) P-WS/P-WW comparisons. The size of the dots represents the number of genes, and different colors show the significance level. Top 30 GO terms, including 10 GO terms for each category: BP, CC, and MF from bottom to top.

The DEGs were divided into three categories, named biological process (BP), cellular component (CC), and molecular function (MF), using Gene Ontology (GO). The results revealed that grafting-responsive genes (identified in P-WW/C-WW) were enriched in cellular lipid biosynthetic/metabolic process, microtubule-based process, and isoprenoid metabolic process in the BP category. In contrast, the DEGs were enriched in the Golgi-associated vesicle in the CC category. Nucleoside-triphosphatase activity, pyrophosphatase, and hydrolase activity were among the most significant GO terms in the MF group (Fig. 5a). Comparison of homografts under WS and WW conditions showed that GO terms were related to carboxylic acid or organic acid biosynthetic process and responses to oxidative stress in BP. In CC the top 10 GO terms were mainly linked to the mitochondria and ribosome. MF contained the DEGs enriched in transporter activity, heme, or tetrapyrrole binding (Fig. 5b). Unlike self-grafted cucumbers, the comparison of heterografts under WS and WW conditions indicated that in BP the DEGs were related to cellular carbohydrate biosynthetic/metabolic process, disaccharide, trehalose, and aspartate process. GO terms in CC were mainly linked to the cell wall, cell periphery, lumen, and cytoskeleton. Motor, hydrolase, and oxidoreductase activities were among the top 10 MF categories (Fig. 5c).

### Visualizing differentially expressed genes related to cell wall and lipid metabolism

**Figure 6:**
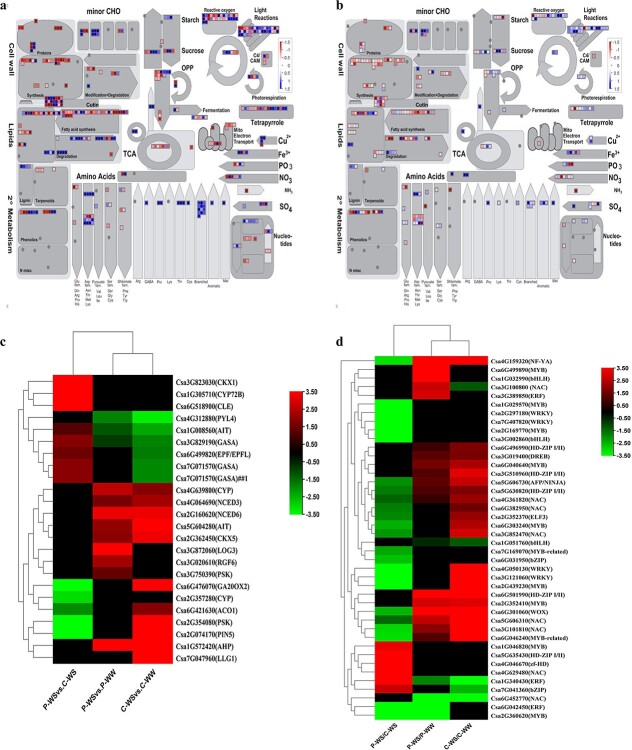
Expression levels of different classes of stress-responsive genes in response to drought stress. Metabolism overview of the identified DEGs related to metabolic pathways in (**a**) P-WS/P-WW and (**b**) C-WS/C-WW. Blue and red colors represent up- and down-regulated genes, respectively. Each square represents an individual transcript. Bins (or sub-bins) are shown as a block. MapMan software was used to classified the genes into different groups. (**c**, **d**) Heatmaps showing a number of identified phytohormones (**c**) and transcription factors (**d**) responsible for drought tolerance. Red and green indicate up- and down-regulation, respectively. Values are shown as log_2_-fold change.

MapMan 3.6.0RC1 software was used to visualize the DEGs into detailed categories. An overview of the metabolism pathway showed that there were 432 and 308 genes in C-WS/C-WW and P-WS/P-WW, respectively, that potentially contributed to the metabolic pathways (Fig. 6). Comparison of homografts and heterografts under WS showed that there were 30 DEGs related to cell wall metabolism, including genes responsible for cell wall proteins [expansin, leucine-rich repeat (LRR)-domain extensin], cell wall synthesis, cell wall modifications [regulatory proteins (*COB*), xylan *O*-acetyltransferase (*XOAT*), endo-β-1,4-mannanase, and pectin lyase], cutin, suberin, and lignin. Cell wall modification genes were highly up-regulated in P-WS/P-WW but down-regulated in C-WS/C-WW. Some of the most up- or down-regulated DEGs are shown in Table 1. Notably, 20 out of 30 cell wall-related genes were up-regulated, while only 9 of them were down-regulated as a response of heterografts to WS compared with homografts. The P-WS/C-WS comparison showed that grafting cucumber onto pumpkin altered the expression of 38 genes linked to lipid metabolism in response to WS. Almost all fatty acid biosynthesis genes were induced in heterografts, including genes coding for ∆7/∆9 fatty acid desaturase (*FAD5/ADS*), fatty acid elongation complex 3-ketoacyl-CoA synthase (*KCS*), and the β chain of the ATP-dependent citrate lyase complex. In contrast, lipid degradation genes were all repressed, except phospholipase A1 (*PC-PLA1*) and α-dioxygenase, which were up-regulated by 2.5 and 3.1, respectively (Fig. 6a and b). Detailed information is provided in Table 1.

**Table 1:** Identified drought stress-responsive DEGs in heterografts and homografts

**Gene ID**	**Description**	**P-WS/P-WW**	**C-WS/C-WW**	**P-WS /C-WS**
**Carbohydrate metabolism**				
**Sucrose metabolism**				
Csa3G168930	Sucrose-specific invertase (CIN)	1.5095458	3.5440083	−1.6607574
Csa4G001950	Sucrose synthase (SUS2)	3.2498798	5.645367	−2.7516963
**Starch metabolism**				
Csa7G213180	Starch branching enzyme (SBE3)	−2.4152193		
Csa5G599830	Glucan, water dikinase (GWD)		1.5394981	−0.9932435
Csa6G072990	β-Amylase (BAM9)	0.6561286	1.3471597	
Csa3G133950	β-Amylase (BAM1)	0.4999263	1.7649453	−1.2041333
**Trehalose metabolism**				
Csa4G028470	Trehalose-6-phosphate phosphatase	2.455504	4.152487	
**Oligosaccharide metabolism**				
Csa1G181330	Galactinol synthase (GOLS1)	2.9800751		
**Nucleotide sugar biosynthesis**				
Csa6G500600	Myo-inositol-1-phosphate synthase	2.314191	1.9062318	
Csa3G434960	Myo-inositol oxygenase	1.3159702		
Csa2G000640	Myo-inositol oxygenase (MIOX2)			5.0860863
**Lipid metabolism**				
**Fatty acid biosynthesis**				
Csa5G177700	3-Ketoacyl-CoA synthase (KCS)		−2.383198	3.722907
Csa4G006100	FAD5/ADS		−2.297169	
Csa4G006160	FAD5/ADS	−2.7915087	−7.949758	7.497645
**Lipid degradation**				
Csa6G490920	Lipase (OBL)	−5.4577723		
Csa4G639890	Lipase (OBL)	−1.7145283		
Csa2G294880	Phospholipase A2 (pPLA2-II)		6.325522	−6.746155
Csa2G372860	Oleosin structural protein		4.887501	−6.275895
Csa1G614650	Phospholipase A1 (PC-PLA1)			2.504306
**Redox homeostasis**				
Csa4G658600	Catalase (CAT1)	3.7495756	2.4154909	
Csa5G179760	Dehydroascorbate reductase (DHAR)	1.2188144	0.9027151	
Csa1G038320	Atypical thioredoxin (ACHT)	1.6192343	1.2288752	
Csa3G874380	Nucleoredoxin	5.2096727	3.521118	
Csa1G013250	NADPH oxidase (Rboh)	2.3208895		1.5053201
Csa5G604150	H-type thioredoxin	1.4112504		
**Cell wall proteins**				
Csa5G636630	α-Class expansin (EXPA5)		−2.5250304	1.7848212
Csa7G050800	LRR-domain extensin (LRX4)		−5.662529	5.1522346
**Cell wall modification**				
Csa4G129040	Regulatory protein (COB)		−5.791909	
Csa3G225840	Regulatory protein (COB)	1.3318733		
Csa6G011700	Xylan *O*-acetyltransferase (XOAT)		1.7431524	
Csa5G648730	Endo-β-1,4-mannanase (MAN5)	5.5644603		
Csa3G624020	Pectate lyase			1.5043529
**Cell wall**				
**Cutin and suberin**				
Csa5G153010	Fatty acyl ω-hydroxylase	−1.8245064	−3.0679615	1.59599
Csa1G340430	Transcription factor (SHN)	−1.811312	−4.438332	3.778865
**Lignin**				
Csa7G431440	Hydroxycinnamoyl transferase (HCT)	1.0359634	3.1313324	−1.7081945

### Visualizing differentially expressed genes related to carbohydrate metabolism and redox homeostasis

Genes encoding carbohydrate metabolism, such as sucrose, starch, oligosaccharide, trehalose metabolism, and nucleotide sugar biosynthesis, were mainly up-regulated in response to drought in both homo- and heterografted cucumbers. Interestingly, starch degradation genes [glucan, water dikinase (*GWD*), β-amylase] were all up-regulated in C-WS/C-WW, while the starch biosynthesis gene (starch branching enzyme) was down-regulated in P-WS/P-WW. The genes coding for sucrose-phosphate synthase, alkaline sucrose-specific invertase (*CIN*), sucrose synthase, trehalose-6-phosphate phosphatase, galactinol, and raffinose synthase were all up-regulated in both homografts and heterografts. All the genes in the nucleotide sugars biosynthesis category were up-regulated in cucumber grafted onto pumpkin under WS (Fig. 6a and b).

Since redox homeostasis is another essential plant response to stressful conditions, we extracted the DEGs related to this phenomenon. There were 16 and 30 DEGs associated with this reaction in P-WS/P-WW and C-WS/C-WW, respectively. Nucleoredoxin and catalase were the two most up-regulated genes in P-WS/P-WW that showed a higher expression level than in homografts (Fig. 6a and b). NADPH oxidase (*Rboh*) was another redox homeostasis-related gene that was differentially expressed in P-WS/P-WW (Fig. 6a and b; Table 1).

### Identification of phytohormones, transcription factors, and protein kinase-related genes under drought stress

It is worth mentioning that we also found many DEGs connected to phytohormone actions in this study. Cucumber grafted onto pumpkin rootstock compared with cucumber grafted onto its own rootstock under WS changed the expression level of genes coding for ABA [abscisic acid transporter (*AIT*), abscisic acid receptor (*PYL4*), 9-*cis* epoxycarotenoid dioxygenase (*NCED3*, *NCED6*), auxin transporter (*PIN*), brassinosteroid hydroxylase (*CYP72B*), and cytokinin dehydrogenase (*CKX1*)] in response to WS. Among various phytohormone-related genes, the signaling peptide sub-bin contained the highest number of DEGs, with seven up-regulated and four down-regulated genes. Cytokinin dehydrogenase and CLE precursor polypeptide were the most up-regulated hormones in heterografted cucumber under WS (Fig. 6c).

We identified 112 DEGs for transcription factors in the P-WS/C-WS comparison, of which, *AP2*/*ERF*, *MYB*, and *WRKY* were the top three transcription factors, with 19, 16, and 10 DEGs, respectively. The rest of the transcription factor-related genes were distributed in various families, in which the *NAC*, *bZIP*, and homeobox families contained more DEGs than the other families. The highest expression level of transcription factor genes was found in the homeobox (*HD-ZIP I*/*II*, *zf-HD*, *HD-ZIP IV*), *NAC*, *MYB*, *bZIP* and *ERF* families (Fig. 6d).

Protein kinase is another group of regulatory proteins involved in the plant’s responses to stress. Surprisingly, all DEGs corresponding to protein kinase were repressed in P-WS/P-WW except one gene, which belonged to the TKL protein kinase superfamily (*RLCK-IXb*), which was vigorously up-regulated. In contrast, in the C-WS/C-WW comparison not only was a larger number of genes expressed differentially, but also their expression intensity was greater than in pumpkin-rooted plants. Some of the identified DEGs linked to protein kinase are listed in Supplementary Table.

### Identification of putative mobile mRNAs in P-WW and P-WS

**Figure 7:**
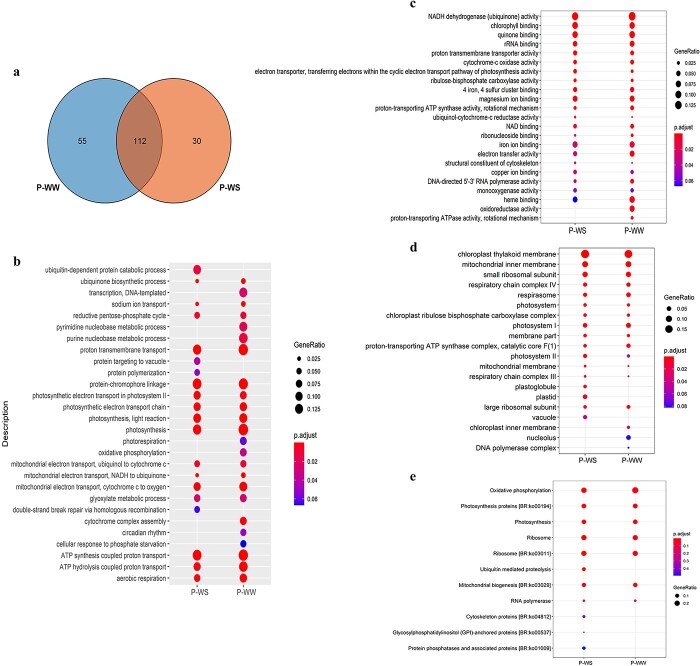
Venn diagram and enrichment analysis of mb-mRNAs identified in heterografts. (**a**) Number of common and specific mb-mRNAs in heterografts under P-WW and P-WS conditions. (**b**) Biological process, (**c**) molecular function, and (**d**) cellular component categories of GO enrichment analysis for identified mb-mRNAs. (**e**) KEGG enrichment analysis of putative mb-mRNAs. The size of the dots represents the number of genes and different colors show significance levels.

We also identified the mb-RNAs that had translocated from pumpkin rootstock to the cucumber scion under WW or WS conditions. The results indicated that there were 167 and 142 mb-mRNAs in heterografts under WW and WS conditions, respectively. In addition, 112 of these mb-mRNAs were translocated from the rootstock to the scion regardless of the WW or WS condition (Fig. 7a). We performed GO and KEGG (Kyoto Enclyclopedia of Genes and Genomes) analyses to investigate which pathways these mRNAs are involved in. Our findings indicated that these mb-mRNAs were mostly related to the ubiquitin, mitochondrial, and photosynthetic processes (such as light reaction, photosystems, and electron transport chain) in the BP category. Chloroplast thylakoid membrane, mitochondria membrane, plastid, and photosystem I and II were in the CC category. In the MF category, GO terms were mostly related to ubiquinone activity, chlorophyll, cytochrome c oxidase activity, photosynthesis, and oxidoreductase activity (Fig. 7b–d). The results of KEGG analysis revealed that the most significant pathways were related to oxidative phosphorylation, photosynthesis proteins, and ubiquitin-mediated proteolysis (Fig. 7e).

### Specific mobile mRNAs in P-WS were related to stress tolerance

**Figure 8:**
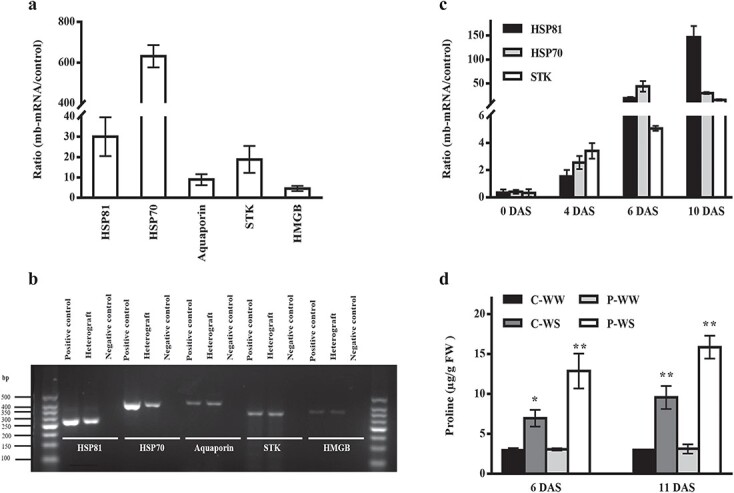
Validation of predicted mb-mRNAs and dynamic expression level of three mb-mRNAs accompanied by proline accumulation. (**a**) Verification of the existence of five mb-mRNAs of pumpkin rootstock in cucumber scion under the P-WS condition using qRT–PCR. The *x*-axis shows the five selected mb-RNAs in P-WS and the *y*-axis indicates the ratio of mRNA abundance in heterografts compared with control (homografts) under drought stress (11 DAS). The samples were taken from cucumber scion grafted onto pumpkin rootstock and the primers were specific for pumpkin without hits in cucumber. (**b**) mb-mRNA verification using RT–PCR analysis. In this experiment, pumpkin and cucumber under WS <<drought stress>> were used as positive and negative control, respectively. (**c**) Dynamic abundance of three verified stress-responsive mb-mRNAs in heterografts under WS <<drought stress>>. P-WW was used as control. (**d**) Proline content in homografts and heterografts in response to WS <<drought stress>>.

We focused on mb-mRNAs that were specifically identified in P-WS or P-WW. The Venn diagram illustrated that 30 and 55 mb-mRNAs were specifically identified in response to WS and WW conditions, respectively (Fig. 7a). Among 30 specific mb-mRNAs for P-WS (strongly expressed in P-WS but with very low abundance in P-WW) we found 14 well-known stress-responsive genes, such as serine/threonine-protein kinase (*STK*), heat shock protein (*HSP81*), 70-kDa heat shock protein (*HSP70*), high mobility group B protein (*HMGB*) and transmembrane water channel protein (aquaporin) (Table 2). Since *HSP70*, *HSP81*, and *STK* had the highest abundances in the leaves of heterografts under WS (Fig. 8a and b), we examined the dynamic expression level of these mb-mRNAs at 0, 4, 6 and 11 DAS. The results showed that the abundance of these mb-mRNAs sharply increased at 6 and 11 DAS but showed a low abundance level in the initial days after WS (Fig. 8c). It is worth noting that we used P-WW as a control to compare the abundance of mb-mRNAs in the cucumber scions heterografts under WS. Since HSP70 has been reported to improve abiotic stress through proline accumulation, we also measured proline content. Interestingly the increase in the expression level of HSP70 was accompanied by proline accumulation at 6 and 11 DAS (Fig. 8d). Our results indicated that there were some mb-mRNAs related to photosynthesis in P-WW and P-WS, some of which are shown in Table 3.

**Table 2:** Drought stress-responsive mb-mRNAs in heterografts (P-WS)

**Gene ID**	**Description**	**GO term**
CmoCh01G015950.1	Glucan endo-1,3-β-glucosidase	Carbohydrate metabolic process, defense response, cell wall
CmoCh04G020740.1	ATP synthase subunit a	ATP synthesis coupled proton transport
CmoCh06G000970.1	DnaJ-like subfamily B member 8	Protein folding
CmoCh06G007160.1	Heat shock protein 81.4	Response to stress
CmoCh07G010280.1	70-kDa heat shock protein (HSP70)	Oxidation–reduction process
CmoCh09G000520.1	High mobility group B protein 2 (HMGB)	Chloroplast
CmoCh12G001440.1	Chaperone protein DnaJ	Protein domain specific binding
CmoCh15G009410.1	α-Glucosidase	Sucrose metabolic process
CmoCh15G012980.1	Transmembrane water channel protein	Transporter activity
CmoCh18G001250.1	Serine/threonine-protein kinase-transforming protein (STK)	Protein phosphorylation
CmoCh18G002910.1	Ubiquitin	Cellular protein modification process
CmoCh16G009140.1	Ubiquitin	Cellular protein modification process
CmoCh03G005460.1	Ubiquitin	Cellular protein modification process
CmoCh14G002590.1	Peroxiredoxin	Peroxidase activity

**Table 3:** Photosynthetic related mb-mRNAs identified in heterografted cucumber

**Gene ID**	**Description**	Stress condition
CmoCh17G009900	Photosystem II CP47 reaction center protein	P-WS
CmoCh15G006560	Photosystem II CP47 reaction center protein	P-WS
CmoCh10G011100	Photosystem II protein D2	P-WS
CmoCh12G006890	Photosystem II protein D1	P-WW
CmoCh00G000540	Photosystem II CP43 reaction center protein	P-WW
CmoCh11G012220	Photosystem I p700 chlorophyll a apoprotein A2	P-WS
CmoCh11G012200	Photosystem I P700 chlorophyll a apoprotein A1	P-WS
CmoCh17G010900	Apocytochrome f	P-WW
CmoCh00G000730	Photosystem I P700 chlorophyll a apoprotein A1	P-WW
CmoCh00G000560	Photosystem I P700 chlorophyll a apoprotein A1	P-WW
CmoCh16G005090	Chloroplast photosystem II light-inducible protein	P-WS
CmoCh15G004580	Photosystem I light-harvesting chlorophyll a/b-binding protein	P-WS
CmoCh11G012210	Photosystem I P700 chlorophyll a apoprotein A1	P-WS
CmoCh00G000550	Photosystem I p700 chlorophyll a apoprotein A2	P-WS

### Verification of DEGs and mobile mRNAs using quantitative real-time PCR and reverse transcription–PCR

In order to verify the result of transcriptome sequencing, 10 stress-responsive genes were selected, and their expression levels were checked using quantitative real-time PCR (qRT–PCR). The high correlation coefficient value (*R*^2^ = .95) between expression levels of selected genes in RNA-seq and quantitative PCR results showed that these values have similar expression patterns with both methods and confirmed the accuracy of our transcriptome sequencing results (Supplementary Fig. S1).

To verify the existence of pumpkin (rootstock) mRNAs in cucumber (scion), which had been identified using transcriptome data, we selected five mb-mRNAs (*HSP81*, *HSP70*, *STK*, *HMGB*, and aquaporin), then checked their abundance in cucumber scion grafted on pumpkin rootstock by qRT–PCR using pumpkin-specific genes. Our results confirmed that all five mb-mRNAs had a much higher expression level in heterografts compared with homografts under WS, especially HSPs, which showed the highest expression level in heterografts compared with the negative control (Fig. 8a). We further examined the mobility of these five mb-mRNAs in heterografts (P-WS) through reverse transcription–PCR (RT–PCR) analysis. cDNA of cucumber scion grafted onto pumpkin rootstock was used as a template to amplify the predicted mobile genes that originally belonged to pumpkin. Pumpkin and homografts were used as the positive and negative control, respectively. Based on agarose gel electrophoresis results, the RT–PCR products of these five mb-mRNAs were detected. Therefore, we confirmed the mobility of these mb-mRNAs by qRT–PCR and RT–PCR analysis (Fig. 8b). It is worth noting that we could verify the existence of both highly expressed (*HSP70* and *HSP81*) and lowly-expressed (*HMGB*) mb-mRNAs in cucumber scion. Therefore, based on the verified mb-mRNAs (with low and high abundance), our results suggest no association between the abundance and movement ability of mRNAs.

## Discussion

In this research, we aimed to investigate the responses of heterografts and homografts to WS. Drought tolerance is a very complicated trait that can be achieved by various modifications at the molecular and physiological levels, such as photosynthesis, lipid metabolism, cell wall modifications, antioxidant enzymes, osmotic adjustment, phytohormones, and especially ABA and ROS such as H_2_O_2_, which can act as signals. Therefore, we did transcriptome sequencing to identify the stress-responsive genes (including mobile and immobile) induced by heterografts and also examined the corresponding physiological modifications, and then compared the responses of homografts and heterografts to WS.

### Pumpkin rootstock changed the expression level of numerous genes in cucumber scion under control or drought stress conditions

We did transcriptome sequencing to understand the molecular modifications induced by grafting in response to WS. As shown in Fig. 4, the huge number of DEGs in P-WW/C-WW suggest the effective control of pumpkin rootstock over cucumber scion. Many of these DEGs have been reported to contribute to abiotic stress tolerance [33], suggesting the possible role of grafting-responsive genes in induction of the constitutive modifications in cucumber scion to improve stress tolerance [34]. After applying WS, the cucumber rootstock seems to be more disturbed by WS. Therefore, most of the genes were up-regulated, and gene expression intensity was mainly greater than that in heterografts. Greater gene expression level in the sensitive species in comparison with tolerant species has been reported in other crops, such as sesame, rice, banana, and barely [35]. A possible reason for this response to drought might be the absence of a robust homeostasis mechanism in sensitive genotypes [35].

### Pumpkin rootstock improves growth condition in cucumber scion by expression of immobile and mobile genes linked to photosynthesis

Drought tolerance is a very complicated trait, and plant responses can widely vary based on the plant genotype, stress severity, and even daytime [26]. In this study, we categorized all transcripts in different groups that have been reported as stress-responsive modifications, then examined their corresponding physiological responses to WS. As we identified several mb-mRNAs coding for photosynthesis-related process (Table 3), we measured some traits related to photosynthesis, such as stomatal conductance, Fv/Fm, chlorophyll contents, and plant height. According to our findings, heterografts could improve the above indices and increase photosynthesis and growth rate under WS compared with homografts (Fig. 1). It seems that pumpkin rootstock is able to improve photosynthesis in the scion by translocating mb-mRNAs (originally belonging to pumpkin) from the rootstock to the scion. The better growth rates of heterografts under both WS and WW conditions could be attributed to the considerable number of edna mb-RNAs associated with photosynthesis in heterografts. These mb-mRNAs were mostly the key components of photosynthesis, such as photosystem I and photosystem II, which help heterografts to improve photosynthesis and growth rate (Table 3).

Stomatal conductance decreased significantly in heterografts and homografts at 6 and 11 DAS, respectively (Fig. 1c). The capability of cucumber scion grafted onto pumpkin rootstock to reduce stomatal conductance earlier than in homografts might lead to better management of transpiration rate, since heterografts showed higher RWC under WS as well as 1 day after recovery (Fig. 1a and b). Higher stomatal conductance in heterografts at the late stage of the drought compared with homografts might provide enough gas exchange to increase photosynthesis under WS. According to previous studies, the tolerant rootstock is able to sense the adverse conditions at the very early stage of stress, and to show the quick response to stress that helps plants to have a better water content at the later stage of adverse soil conditions [9, 17, 36].

### Lipid metabolism-related genes assist heterografts in maintaining cell membrane integrity under drought stress

Modifying the expression of genes linked to the lipid metabolism pathway is one of the critical responses to stress conditions. Fatty acids are essential components of the cell membrane and have been reported to be affected by environmental threats. However, fatty acid desaturase enzymes (FADs) help the cell membrane to retain its function under stress by producing unsaturated fatty acids [37]. Overexpression of two FAD genes (*FAD3* or *FAD8*) in tobacco improved its drought tolerance [38]. These findings are consistent with our results, in which FAD was strongly up-regulated (log_2_-fold change 7.49) in pumpkin-rooted plants compared with homografts under WS (Table 1). Phospholipase enzymes were also among the strongly repressed genes in heterografts under WS. Hong *et al.* [39] reported that higher activity of phospholipase D could confer stress resistance only in the early stage of the stress, while its continuous up-regulation at the late stage of drought increased sensitivity to stress. This result might be a reasonable explanation for the strong down-regulation of phospholipase A2 in heterografts under WS, as our sampling time for RNA sequencing was at the late stage of WS (Table 1). The higher percentage of electrolyte leakage and lipid peroxidation (MDA) in C-WS compared with P-WS was strong evidence to prove that homografts suffered from the dehydrated condition more than heterografts (Fig. 2f and g). Membrane degradation indicating cell injury, which eventually reduces the water content and the plant’s ability to survive under environmental stresses [39]. Besides, our results showed that almost all lipid biosynthesis genes were up-regulated and the lipid degradation genes were down-regulated in heterografts. A combination of transcriptome and physiological (lower REL and MDA) results suggests that heterografts are able to maintain cell membrane integrity under WS by reducing the expression level of lipid degradation genes as lipids are major components of the cell membrane.

### Differentially expressed genes potentially linked to cell wall metabolism pathways

Cells sense environmental stimuli by transducing signals from the cell wall to inside the cell. Therefore cell wall-related genes play essential roles in the signaling pathway under abiotic stresses [40]. Our results indicated that 30 genes that potentially contributed to cell wall metabolism were differentially expressed in heterografts compared with homografts under WS. Among them, genes coding for cell wall proteins (LRR-extensin and α-class expansin proteins) and suberin and SHN-type cutin and suberin biosynthesis transcription factors were expressed at a greater level than others (Fig. 6a and b; Table 1). Zhao *et al*. showed the essential role of LRR-extensin proteins in the maintenance of cell wall integrity and abiotic stress tolerance [41]. Another study reported that overexpression of the wheat expansin gene (*TaEXPA2*) led to retention of water content under drought conditions and increased drought tolerance in transgenic tobacco [42]. Aharoni *et al.* showed that overexpression of SHN-type proteins (belonging to the AP2/EREBP transcription factor superfamily) in *Arabidopsis* leads to improvement of drought tolerance by a reduction in transpiration rate through reducing the number of stomata [43].

### Heterografts and homografts might employ different mechanisms to exert osmotic adjustment using differentially expressed genes linked to carbohydrate metabolism

Accumulation of various kinds of carbohydrates is one of the usual responses to WS. Soluble sugars participate in osmotic regulation to maintain cell turgor [44]. To investigate the correlation between transcriptome and physiological responses to WS, we measured the TSS and starch contents, as we identified 42 genes related to carbohydrate metabolism in P-WS/P-WW and 62 genes in C-WS/C-WW (some of the DEGs are shown in Table 1). We found that both grafted materials increased TSS and reduced starch as a response to WS. However, it seems that heterografts increased TSS by reducing starch biosynthesis, while homografts achieved this enhancement by starch degradation. Since homografts had an excess amount of starch under WW conditions (compared with heterografts), they degraded starch to sugars in response to WS, which will be subsequently used in the osmotic adjustment process. In contrast, heterografts reduced starch biosynthesis to maintain its substrates (sugars) at a high level (Fig. 2d and e). In support of this statement, we found that homografts (C-WS/C-WW) up-regulated the starch degradation genes (*GWD*, β-amylase). At the same time, heterografts down-regulated the starch biosynthesis gene (starch branching enzyme) in response to WS (Table 1). Briefly, our results suggested that heterografts and homografts use different strategies for osmotic adjustment: homografts enhance sugar content by degradation of starch, whereas increasing the sugar content in heterografts might be a result of reduction in starch biosynthesis. Since starch is synthesized from simpler carbohydrates such as sucrose, glucose, and hexose and these metabolic pathways need energy, reduction in starch biosynthesis in heterografts not only maintains a better energy level for critical metabolic pathways under stress but also allows starch precursors to contribute to osmotic adjustment instead of starch biosynthesis. These results are consistent with other reports in maize and rice under severe abiotic stresses and in cucumber grafted onto pumpkin under Ca(NO_3_)_2_ stress [45, 46].

### Redox homeostasis is maintained in heterografts by mobile and immobile mRNAs

Maintenance of redox homeostasis in a proper state is one of the key responses to abiotic stresses, which helps plants to protect their cells through scavenging the extra ROS by increasing antioxidant enzyme activities. Our results demonstrated that nucleoredoxin (*NRX*) and *CAT* were strongly expressed in heterografts under WS (Table 1). NRX1 protects the ROS scavenging capacity of CAT and leads to efficient activity of this antioxidant enzyme in protecting plants against abiotic stress conditions [47]. In addition to these immobile mRNAs, we also identified three mb-mRNAs in P-WS that were related to two antioxidant enzymes (thioredoxin and peroxidase). Therefore, we assessed the activity of three antioxidant enzymes—CAT, SOD, and POD—to investigate the capacity of pumpkin rootstock to protect plants against WS. Pumpkin-grafted plants had a greater capacity to mediate the adverse effects of WS using antioxidant enzymes compared with self-grafted cucumbers (Fig. 2a–c). It seems that pumpkin rootstock is able to improve the antioxidant capacity of the scion against WS by expression of both immobile and mobile mRNAs. Grafting with a tolerant rootstock has been reported in many other crops, such as tomato [48], tobacco [49], luffa [4], and sweet orange [50], to confer efficient antioxidant capacity to protect plants under adverse conditions.

### Differentially expressed genes related to phytohormones, transcription factors, and protein kinases

Phytohormones and transcription factors are two components of the signal transduction network that regulate the response of plant species to WS. In this study, the identified DEGs coding for phytohormones were related to several hormones, such as ABA, auxin, cytokinin, ethylene, gibberellin, strigolactone, brassinosteroid, and signaling peptides (Fig. 6d). AIT, NCED3, NCED6, neoxanthin cleavage protein, and abscisic acid hydroxylase were all up-regulated in heterografts under the WS condition (Fig. 6d). The P-WS/C-WS comparison showed that the hormonal class of signaling peptides had the largest number of genes that are known to play an important role in sensing WS [51]. CLE 9, 10, and 25 (categorized as signaling peptides) were reported to be involved in stomatal closure and WS improvement in *Arabidopsis* [52]. Cytokinin dehydrogenase is another plant hormone-related gene that is up-regulated at high levels in cucumber grafted onto pumpkin under drought conditions. Previous studies demonstrated that ectopic expression of the *CKX* (cytokinin oxidase/dehydrogenase) gene of tobacco [53] and alfalfa [54] in *Arabidopsis* increased drought and salt tolerance, respectively, by modifying the shoot/root ratio and reducing ROS by increasing antioxidant enzymes.

Transcription factors have been reported to contribute to the regulation of plant responses to WS through acting on downstream stress-related genes. Our results indicated that WS changed the expression level of many transcription factors, including *AP2*/*ERF*, *bHLH*, *MYB*, homeobox, *NAC*, *C2C2*, *NF-Y*, *WRKY*, *bZIP*, and many others. Comparing cucumber grafted onto pumpkin and cucumber rootstocks showed that *HD-ZIP I/II* and *zf-HD*, which belonged to the homeobox transcription factor family, had the highest expression level in response to WS (Fig. 6e). *AtHB7*, a component of the *HD-ZIP* transcription factor family, has been shown to have a vital role in response to WS in *Arabidopsis* in overexpression experiments [55, 56]. *ABR1*, a member of the *ERF* transcription factor family, was strongly repressed in the present study. This transcription factor has been previously reported to be expressed under salt and WS as a negative regulator of stress-responsive genes [57]. Based on these findings, it seems that pumpkin rootstock also uses this transcription factor as a negative regulator to induce drought tolerance in the cucumber scion.

The negative or positive modulating activity of protein kinases in response to different stress conditions has been reported previously. The TKL protein kinase superfamily bin contained the largest number of genes that were expressed differentially in heterografts in response to severe water deficit in our study (Supplementary Table S1). Transgenic and mutant experiments using *Arabidopsis* demonstrated that the loss of function of *AtPR5K2*, a member of the receptor-like protein kinase family, increased plant tolerance to water deficit by negative adjustment of ABA, while overexpression lines became supersensitive to stress [58].

### Putative mobile mRNAs involved in mitochondrial- and chloroplast-related metabolism

In the grafting experiments, there is a possibility for translocating the mRNAs from rootstock to scion or vice versa. Therefore, we attempted to identify the putative mb-mRNAs in the cucumber scion that originally belonged to the pumpkin rootstock. Our results revealed that the putative mb-mRNAs were mostly related to the chloroplast, mitochondria, and their corresponding functions. Our findings showed that there were various GO terms linked to the mitochondrial electron transport chain in the BP, CC, and MF categories (Fig. 7b–d), suggesting that one of the possible ways to improve drought tolerance by pumpkin is to translocate mitochondrial-related transcripts to the scion to maintain mitochondrial function in the cucumber scion. Previous findings have revealed the significant role of mitochondria under biotic or abiotic stresses [59, 60]. This organelle is able to collaborate with chloroplasts, the nucleus, and the cytosol and to employ mechanisms to inhibit the excessive reduction of chloroplast under WS [61].

KEGG analysis indicated that ubiquitin-mediated proteolysis was one of the significant pathways in drought stress, in which these putativemRNAs were enriched, but not in the control condition (Fig. 7e). Ubiquitination is a process that leads to inactivation of the target proteins by ubiquitin, which has been shown to play important roles in ABA signaling, stomatal aperture, and drought tolerance improvement in rice [62], *Arabidopsis* [63, 64], and other crops [65]. In addition to the critical role of ubiquitin in WS tolerance, a previous study proved the mobility of some ubiquitin-related mRNAs (NADH-ubiquinone oxidoreductase B18 subunit or *AT2G02050*) across graft junctions [66]. Oxidative phosphorylation (OXPHOS) was the most significant pathway in KEGG analysis (Fig. 7e). This process, which takes place in the inner membrane of mitochondria, has been reported to be involved in plant responses to abiotic stresses, maintaining the stable state of oxidation–reduction of the cells and providing energy (ATP) for various metabolic processes [67, 68]. A previous study revealed that tolerant species preserved mitochondrial structure and increased proteins linked to the OXPHOS pathways. Since some metabolic pathways, such as glutamine or alanine metabolism, increased in tolerant species under salt stress, OXPHOS helps plants to provide a sufficient amount of energy for these cellular processes [69].

### Mobile mRNAs in P-WS contribute to drought stress tolerance in heterografts

It seems that the pumpkin rootstock exerts its control on the cucumber scion not only by changing the expression level of cucumber genes but also by translocation of a number of mb-mRNAs to the scion, which might further play essential roles directly or even act as a signal to activate other critical pathways for WS adaptation. In agreement with this, we identified a number of stress-responsive mb-mRNAs in P-WS that were related to carbohydrate metabolism, response to stress, peroxidase activity, and maintenance of protein structures (Table 2). *HSP*s, *HMGB*, chaperon protein *DnaJ*, α-glucosidase, and *STK* were among the specific mb-mRNAs for WS that have been reported to be involved in abiotic stress tolerance [19, 70, 71]. Besides the mb-mRNAs identified to be linked to stress tolerance, we also identified various mb-mRNAs associated with photosynthesis in P-WW and even P-WS (Table 3), suggesting the potential role of these mb-mRNAs in photosynthesis and growth improvement under WW and WS conditions.

### Severity of drought stress affects the movement of stress-responsive mobile mRNAs

We selected the top three confirmed mb-mRNAs (*HSP81*, *HSP70*, and *STK*) based on the qRT–PCR validation results (Fig. 8a and b), then examined their abundances at different days after stress in P-WS and P-WW. Interestingly, our results demonstrated that the expression levels of these stress-responsive mb-mRNAs were increased by increasing the exposure time to WS (Fig. 8c). These findings suggested that the severity of WS could affect the movement ability of these three stress-responsive mb-mRNAs. *HSP70*, the most up-regulated mb-mRNA, was involved in the induction of abiotic stress tolerance by protecting the cell, removing the oxidized protein, maintaining the protein structures, and cell homeostasis under stressful conditions [19]. Overexpression of HSP in rice led to the induction of drought tolerance by reducing lipid peroxidation, cell membrane protection, and increasing proline contents [72]. Another study provided evidence to show that HSP70 is able to accumulate in cucumber grafted onto luffa, which assists heterografts in removing oxidized proteins and subsequently contributes to conferring stress tolerance on heterografts [18]. Therefore, in agreement with these studies, our results suggest that the accumulation in the scion of heterografts of two mobile HSPs (HSP70 and HSP81), which act as molecular chaperones, could protect plants by increasing proline contents (Fig. 8d), reducing lipid peroxidation, and maintaining cell membrane integrity under WS.

### Abscisic acid, hydrogen peroxide and HSP70 are involved in the drought stress signaling pathway

ABA is a well-known stress-responsive phytohormone that is pivotal for plants’ adaptation to abiotic stresses, mainly through induction of stress-responsive genes and stomatal closure [73]. Our results recorded an earlier ABA accumulation (at 6 DAS) in the leaves of heterografts, which was accompanied by ABA and H_2_O_2_ accumulation in xylem (below the graft union). However, compared with heterologous grafting, the late leaf ABA accumulation in homografts might reflect the late stress sensing and signaling in cucumber. Xylem ABA accumulation could not increase leaf ABA in homografts (at 6 DAS). Interestingly, H_2_O_2_ showed a similar enhancing pattern in the xylem and leaf in response to WS. It seems that root-sourced H_2_O_2_ in heterografts could act as a high-speed signal and transduces the stress signals to the upper parts of the plants. Consequently, earlier leaf ABA accumulation could induce earlier WS responses in heterografts, such as earlier reduction in stomatal conductance or earlier proline accumulation. Additionally, translocation of known stress-responsive mRNAs such as HSP70 from pumpkin rootstock to cucumber scion might indicate the long-distance control of of the scion by the rootstock to induce drought tolerance through proline accumulation. Our findings, along with previous reports, suggest crosstalk between ABA, H_2_O_2_ and HSP70, which are involved in earlier WS sensing and signaling in pumpkin-grafted plants. In agreement with our results, previous studies have reported the role of ABA and H_2_O_2_ as long-distance signals under stressful conditions [9, 14, 18]. However, some studies proposed other high-speed long-distance signals, such as peptides [51], ROS (H_2_O_2_), or even hydraulic signals (1.6 cm/s) [32].

**Figure 9:**
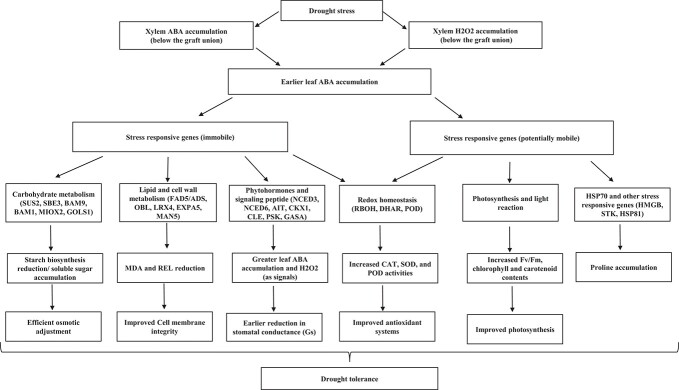
Schematic model of pumpkin-induced drought tolerance in cucumber scion.

In conclusion, our findings provided physiological and transcriptomic evidence for the claim that pumpkin rootstock is able to induce drought tolerance in heterografted cucumbers. According to our results, it seems that the pumpkin rootstock exerts its control over the cucumber scion by earlier ABA accumulation in the leaves, which is accompanied by H_2_O_2_ and ABA accumulation in the xylem below the graft union (pumpkin-sourced signals). The leaves of heterografts sense the WS earlier than homografts, leading to earlier and greater ABA accumulation in the leaves. ABA, as a key phytohormone for stressful conditions, induces drought tolerance modifications in heterografts, such as effective osmotic adjustment, maintaining cell membrane integrity, improving antioxidant systems, and reducing stomatal conductance. In addition to earlier sensing and signaling in heterografts, translocation of the mb-mRNAs linked to WS tolerance (such as HSP70) is more evidence that pumpkin rootstock induces drought tolerance in cucumber scion. Proline accumulation might be a result of increases in HSP70, as this chaperon has been reported to be able to improve drought tolerance by increasing proline content. Besides, in agreement with a recent study [29], enrichment analysis revealed that a considerable number of putative mb-RNAs were enriched in photosynthesis-related reactions such as photosystem II, the light reaction, the chloroplast membrane, and chlorophyll under both well-watered and WS conditions, which could be attributed to the enhanced photosynthesis in heterografts (Fig. 9).

## Materials and methods

### Plant grafting and drought stress treatment

Two kinds of grafted materials were prepared: cucumber (*C. sativus* ‘EC1’) grafted onto its own rootstock and cucumber grafted onto pumpkin (*C. moschata* ‘GMM No. 519’). First, a timetable was designed for sowing cucumber (scion and rootstock) and pumpkin (rootstock) seeds at the appropriate time, allowing us to graft when the rootstock was vigorous enough (Supplementary Table S2). Pumpkin and cucumber seeds, which had been soaked in water for 7 and 3 hours respectively, were sown in peat/vermiculite (3:1, v/v) substrate according to the designated timetable. Hole insertion grafting was performed when the first true leaf came out. Then, both homografts and heterografts were covered with transparent plastic to maintain the humidity at 90%–95% and temperature at ~25–28°C to accelerate the healing process and avoid water loss. One week later, when the grafted plants had survived and the graft junction healed, healthy and uniform plants with similar size were chosen and transferred to pots. Each pot contained one plant and the same amount of substrate with similar water content, 80% of field capacity (FC), determined by the weight difference between dry and wet soil. Thirty-six days after grafting, the grafted plants (C and P) were divided into WW (control condition) and WS (drought stress condition) groups. The water content of WW plants was held as before (80% of FC), while in WS plants water availability was gradually reduced over 11 days as previously described [6]. We weighed all the pots every day and maintained water content at a specific amount for each day: 70%, 60%, 50%, 40%, and 30% of FC for days 1, 2, 3, 4, and 5 of WS, respectively. Then the water availability of the WS group was kept to 30% of FC from Day 5 to Day 11. Weighing the pots helped us reduce water fluctuations as we added a sufficient amount of water to keep a specific amount of water in the soil on each day. The homograft (cucumber as scion and rootstock) and heterograft (cucumber as scion and pumpkin as rootstock) were named C-WW and P-WW, respectively, under well-watered conditions and C-WS and P-WS under drought stress treatment. All grafted materials were grown in a glasshouse at BaiMa Cucumber Research Station of Nanjing Agricultural University, Nanjing, China.

### Stomatal conductance, Fv/Fm, pigment content, and relative water content determination

At 11 DAS, an LI-6400 (Li-Cor, Lincoln, NE, USA) was used to determine gas exchange capacity and Fv/Fm under WW and WS conditions. The topmost expanded leaves were selected to perform this measurement between 10 and 12 a.m. The same samples were used for pigment content (chlorophyll a and b and carotenoid) determination using the Arnon [74] and Soto-Zamora [75] methods. The RWC of the same leaf was examined using leaf disks as described previously [76] at 0, 4, 6, and 11 DAS.

### Evaluation of physiological indices

The topmost leaf samples were taken dynamically at 0, 4, 6, and 11 DAS and 1 day after rewatering (recovery). There were three replications, and each replicate contained at least three plants. The harvested samples were immediately frozen in liquid nitrogen then stored in a refrigerator at −80°C until used for the relevant experiments. REL was estimated using an electrical conductivity method following a protocol described before [77]. MDA, H_2_O_2_, proline, starch, TSS contents and SOD, POD, and CAT activities were all examined using kits provided by Nanjing Jiancheng Bioengineering Institute, following the instructions that were provided in the kits. The ABA content of leaves and xylem tissue was determined at 6 and 11 DAS. Endogenous plant hormones were extracted, and then an Agilent 1290 high-performance liquid chromatograph was connected to an AB QTRAP 6500 mass spectrometer to determine endogenous ABA. The internal standard was added to the extract to correct the test results. The xylem below the graft union was collected at 6 and 11 DAS using sterile tweezers and immediately frozen in liquid nitrogen for ABA and H_2_O_2_ measurements.

### RNA-seq analysis

Twelve leaf samples were collected, including C-WW, P-WW, C-WS, and P-WS, with three biological and three technical replicates. The TRIzol method was used to extract total RNA from leaf samples. RNA integrity was assessed using the RNA Nano 6000 Assay Kit of the Bioanalyzer 2100 system (Agilent Technologies, CA, USA). Sequencing libraries, which were generated using the NEBNext^®^ Ultra™ RNA Library Prep Kit for Illumina^®^ (NEB, USA), were sequenced on an Illumina Novaseq platform by Novogene Company (Shanghai, China), and 150-bp paired-end reads were generated. The reads contained adapters, poly N and low-quality reads were eliminated from raw data to achieve clean data (clean reads). All the analyses were based on clean data with high quality. Paired-end clean reads were aligned to the ‘Chinese Long v2’ cucumber reference genome using Hisat2 v2.0.5 [78]. Gene expression levels were estimated by calculating the fragments per kilobase million of each gene using feature Counts v1.5.0-p3 [79]. The identification of DEGs was done using the DESeq R package. The criteria for screening the significantly expressed genes were *P* < .05 and |log2(FoldChange)| > 1. GO and KEGG enrichment analysis of DEGs was performed by clusterProfiler based on the hypergeometric distribution [80]. To evaluate the function of each DEG, they were compared with the *Arabidopsis thaliana* database (https://arabidopsis.org). Then MapMan software was used to classify the DEGs into more detailed categories and visualize them in diagrams.

### Identification of mobile mRNAs in scion

We performed identification of mb-mRNAs as described previously [30]. As there are some mismatches and single-nucleotide polymorphisms (SNPs) between the cucumber variety ‘Chinese Long’ published reference genome and the cucumber variety that we used for grafting (*C. sativus* ‘EC1’), we first did genome resequencing of the scion (‘EC1’) and reconstructed the reference genome by mapping these data against the published ‘Chinese Long’ reference genome following the procedure that was recently reported with some modifications. The paired-end reads from transcriptome sequencing of either P-WS or P-WW were mapped against the reconstructed reference genome (‘EC1’). Because the samples were from the scion and the reference genome was also constructed based on the scion, the unmapped reads could have originated from pumpkin rootstock. Therefore, we used the BlastN tool to search against the pumpkin reference genome [*C. moschata* (Rifu) genome]. The reads that were matched to the pumpkin reference genome were considered to be candidate mb-mRNAs. Then we found the false-positive genes and removed them from the candidate mb-mRNAs. The unmapped reads of homograft data against the ‘EC1’ genome that were matched to the pumpkin reference genome were considered to be false-positive mb-mRNAs.

### Quantitative real-time PCR

Total RNA extraction was done using leaf samples collected from homo- and heterografted cucumbers at 11 DAS. The PrimerScript RT reagent kit with the gDNA Eraser kit (TaKaRa) was used to synthesize cDNA. Then the relative expression level of several stress-responsive genes was examined using SYBR Premix Ex Taq™ Kit (TaKaRa) in a iQ1 Real-Time PCR System (Bio-Rad). The data were calculated by the 2^-ΔΔCt^ method. The final value was calculated as an average of triplicate reactions. The Ct value of Cs-actin was used to normalize the Ct value of each gene. The list of primers is provided in Supplementary Table S3.

### Verification of mobile mRNAs using qRT–PCR and RT–PCR

We selected five mb-mRNAs to confirm their movements from the rootstock to the scion by qRT–PCR and RT–PCR. The key point for mobility confirmation of a specific gene is the primer sequence, which should be specific for the tissue of origin (in this study the rootstock) with no hits in the destination tissue (in this study the scion). Mobility validation can be achieved by high expression of the mb-mRNA in heterografts but no expression or a very low expression level in homografts. We also performed RT-PCR to further confirm the existence of the selected mb-mRNA in the scion by visualizing the corresponding PCR products on 2.5% agarose gel. Pumpkin and cucumber leaves under stress were used as the positive and negative control, respectively.

## Statistical analysis

We designed the experiment as a completely randomized design with three biological and at least three technical replications. The data were statistically analyzed using ANOVA and Tukey’s test.

## Supplementary Material

Web_Material_uhab033

## Data Availability

The RNA-seq data used in this study have been deposited in the NCBI public database (SRA accession: PRJNA720243).
