# Supplementary material for: Long-distance control of the scion by the rootstock under drought stress as revealed by transcriptome sequencing and mobile mRNA identification
Source: Hortic Res. 2022 Jan 19;9:uhab033. doi: 10.1093/hr/uhab033 (PMC8854630; doi:10.1093/hr/uhab033)

**Supplementary Table S1-** differentially expressed genes associated with protein kinases

| **BinCode** | **BinName** | **id** | **P-WS/C-WS**  **(log_2_foldchange)** |
| --- | --- | --- | --- |
| **TKL** |  |  |  |
| **18.4.1.30** | TKL protein kinase superfamily (MAP3K-RAF) | kgn60267 | -1.23527841 |
| **18.4.1.37.1** | TKL protein kinase superfamily (RLCK-VIIa) | kgn60448 | -1.701436183 |
| **18.4.1.37.1** | TKL protein kinase superfamily (RLCK-VIIa) | kgn51317 | -7.021026908 |
| **18.4.1.2** | TKL protein kinase superfamily (LRR-II) | kgn53203 | -1.242515794 |
| **18.4.1.37.1** | TKL protein kinase superfamily (RLCK-VIIa) | kgn55385 | -2.190810236 |
| **18.4.1.12** | TKL protein kinase superfamily (LRR-XII) | kgn50353 | -1.423804319 |
| **18.4.1.35** | TKL protein kinase superfamily (RLCK-V) | kgn62491 | 0.721515736 |
| **18.4.1.1** | TKL protein kinase superfamily (LRR-I) | kgn51709 | -0.667388015 |
| **18.4.1.10.2** | TKL protein kinase superfamily (LRR-Xb) | kgn63699 | -1.19364489 |
| **18.4.1.3** | TKL protein kinase superfamily (LRR-III) | kgn60492 | -1.375051489 |
| **18.4.1.31.2** | TKL protein kinase superfamily (MLK-II) | kgn60091 | -0.969771316 |
| **18.4.1.15** | TKL protein kinase superfamily (LRR-XV) | kgn66180 | -1.605288684 |
| **18.4.1.37.1** | TKL protein kinase superfamily (RLCK-VIIa) | kgn47532 | 0.551362326 |
| **18.4.1.36** | TKL protein kinase superfamily (RLCK-VI) | kgn56310 | -0.678932869 |
| **18.4.1.37.1** | TKL protein kinase superfamily (RLCK-VIIa) | kgn47146 | -0.721912932 |
| **18.4.1.30** | TKL protein kinase superfamily (MAP3K-RAF) | kgn46154 | -0.829290518 |
| **CMGC** |  |  |  |
| **18.4.3.1.8** | CMGC protein kinase superfamily (CDK9) | kgn46735 | -1.228476275 |
| **18.4.3.1.8** | CMGC protein kinase superfamily (CDK9) | kgn46736 | -2.440597541 |
| **CAMK** |  |  |  |
| **18.4.5.3** | CAMK protein kinase superfamily (SnRK3) | kgn55436 | -0.979156613 |
| **18.4.5.1.3** | CAMK protein kinase superfamily.SNF1-related SnRK1 kinase | kgn63486 | -1.177612751 |
| **18.4.5.3** | CAMK protein kinase superfamily (SnRK3) | kgn49422 | -0.966580478 |
| **18.4.5.4** | CAMK protein kinase superfamily (CDPK) | kgn44377 | -0.832143291 |
| **AGC** |  |  |  |
| **18.4.6.5** | AGC protein kinase superfamily (AGC-VIII) | kgn46933 | -0.858298617 |
| **18.4.6.4** | AGC protein kinase superfamily (AGC-VII/NDR) | kgn49490 | -0.894954124 |
| **18.4.6.5** | AGC protein kinase superfamily (AGC-VIII) | kgn43384 | -3.211050707 |
| **atypical protein kinase familIies** | | |  |
| **18.4.7.4** | Atypical protein kinase families (ABC1) | kgn50753 | -1.02476462 |
| **18.4.7.4** | Atypical protein kinase families (ABC1) | kgn62876 | -0.934822385 |
| **18.4.7.1** | Atypical protein kinase families (PIKK) | kgn43836 | -0.531605337 |
| **STE** |  |  |  |
| **18.4.2.4** | STE protein kinase superfamily (MAP3K-WNK) | kgn47215 | -1.335585504 |
| **18.4.2.2** | STE protein kinase superfamily (MAP3K-MEKK) | kgn63238 | -2.256015179 |
| **18.4.2.2** | STE protein kinase superfamily (MAP3K-MEKK) | kgn48531 | -1.658575978 |

**Supplementary Table S2-** time schedule for scion and rootstock sowing

| **Rootstock (sowing date)** | **Scion (sowing date)** | **Grafting date** |
| --- | --- | --- |
| Pumpkin (2019-5-17) | Cucumber (2019-5-20) | C-P (2019-5-29) |
| Cucumber (2019-5-14) | Cucumber (2019-5-20) | C-C (2019-5-29) |

**Supplementary Table S3-** primes sequences used for qRT-PCR and RT-PCR experiments

| **Gene id** | **Forward** | **Reverse** |
| --- | --- | --- |
| Csa4G614230 | 5' TCAGAGTTTTTAGCAAAGGTGG 3' | 5' AAGCGAAGGAGCGGAGTT 3' |
| Csa4G045040 | 5' GGTGGCATACTTCATCGCT 3' | 5' GGTTGACACTGCTTGCTCTT 3' |
| Csa2G360620 | 5' CACTATTGCTCGGTTGCTTCC 3' | 5' ATTGTTGGTGGTGCTGTTGTTC 3' |
| Csa2G035350 | 5' ACCTGGAAGAACCGACAACGAG 3' | 5' TATGAGAGGCTTGTGAGTGTTGGG 3' |
| Csa6G301060 | 5' TTCCCCAACTACTCCCACAGC 3' | 5' CTCATCGCCTCCCATCCAC 3' |
| Csa2G074170 | 5' CTACGCTTACCAACGCCCT 3' | 5' TCTTCACTCACAACCCCCG 3' |
| Csa6G486790 | 5' ATTCACTTCTCGTCTTCATCGTC 3' | 5' AAAGGTGGGGAAGAAAGAGAA 3' |
| Csa6G452770 | 5' ATGACCAGAAGTTATCTAATCCGCT 3' | 5' ATCAACTTCCATCTTCAACTGCC 3' |
| Csa4G050130 | 5' GAAGGCTGTCAAACATAGCAACCA 3' | 5' TGCTTGAGAAGAGGGGTTAGAGTTT 3' |
| Csa7G222870 | 5' AAGACACCCATTTCATTTATTCCTG 3' | 5' GCATTAGGCAGTTTCTTCTCTTCTT 3' |
| CmoCh06G007160 (HSP81) | 5' CTTATTAGCAACGCCTCAGATG 3' | 5' CCAACTCCGAACTGTCCAA 3' |
| CmoCh07G010280 (HSP70) | 5' CACCGTTCCTGCTTATTTCA 3' | 5' GTCCTCTTAGCCCTCTCACAC 3' |
| CmoCh09G000520 (HMGB) | 5' TTATCATCCCCCCTTCCCT 3' | 5' CGTATCTCTCCACACCATCCA 3' |
| CmoCh15G012980 (Aquaporin) | 5' ACTCCGAAAGACAGCCAATC 3' | 5' CTTCTCAAAACCCTTCACGAC 3' |
| CmoCh18G001250 (STK) | 5' ATTTGGTGTCGTCTGGTGC 3' | 5' TCCTGCTGATGGAGGTATTTT 3' |

**Supplementary Fig. S1:** validation of RNA-seq results by qRT-PCR.


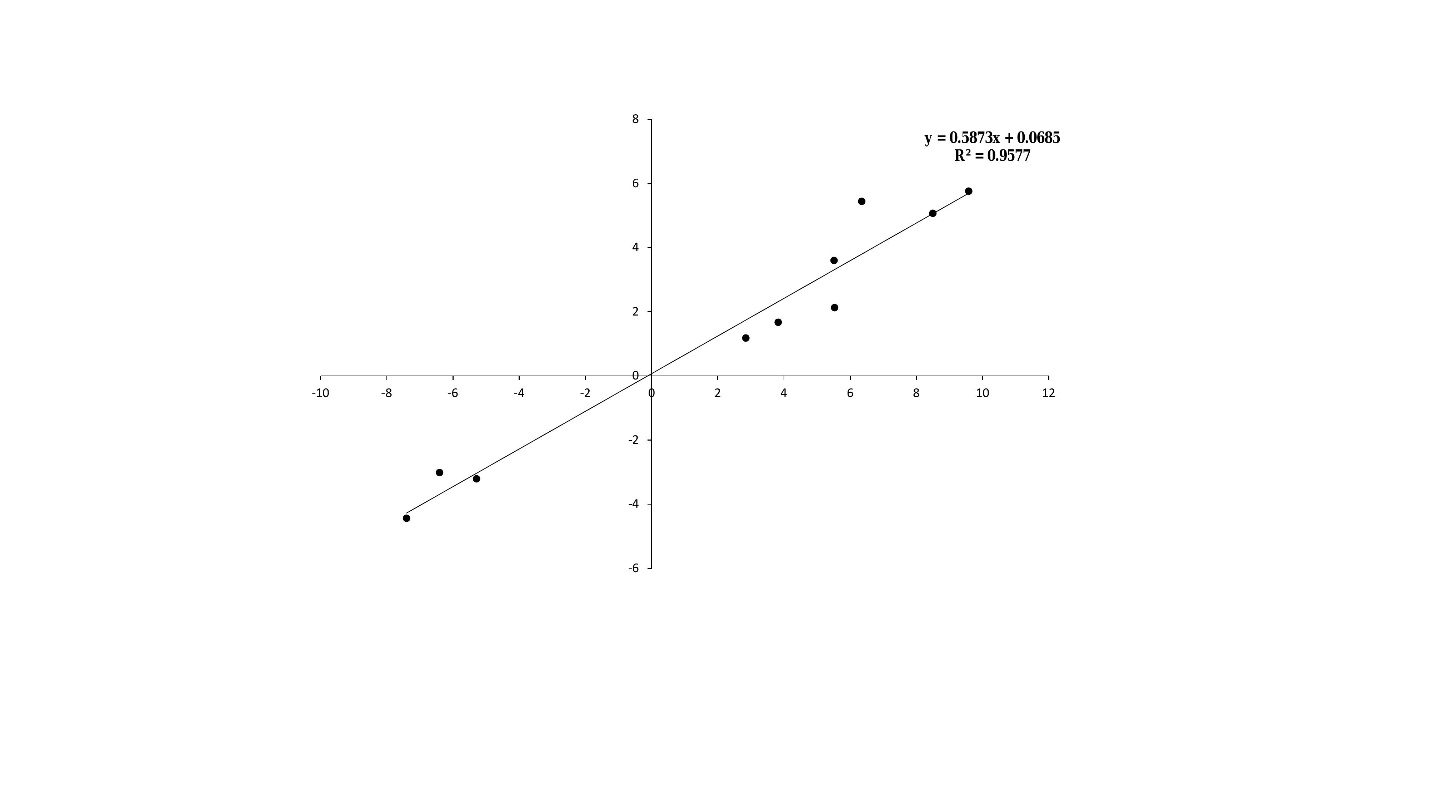

Supplement: Web_Material_uhab033 [file web_material_uhab033.docx]
